# Acute respiratory distress syndrome subphenotypes: the translational path from pathobiology to precision medicine

**DOI:** 10.3389/fphar.2026.1947054

**Published:** 2026-10-20

**Authors:** Han Shao, Tingting Bai, Tongtong Zhang, Yanyan Qiao, Yanyan Li

**Affiliations:** Department of Respiratory and Critical Care Medicine, Tangdu Hospital, The Fourth Military Medical University, Xi’an, China

**Keywords:** acute respiratory distress syndrome, biomarkers, heterogeneity, pathobiology, precision medicine, subphenotypes

## Abstract

Acute respiratory distress syndrome (ARDS) is a highly heterogeneous critical illness syndrome lacking effective targeted therapies, partly due to the diverse patient population. Subphenotype research aims to identify homogeneous patient subgroups sharing common biological, physiological, or clinical characteristics, enabling prognostic and predictive enrichment. This review systematically summarizes current ARDS subphenotype research, from pathobiological mechanisms to multidimensional identification based on etiology, imaging, biomarkers, respiratory mechanics, and transcriptomics. We highlight evidence for differential prognoses and treatment responses across subphenotypes, including variable effects of neuromuscular blockade, corticosteroids, and prone positioning. We further discuss challenges and future directions in translating subphenotype classification into bedside practice. This article provides a theoretical framework and practical guidance for advancing precision medicine in ARDS.

## 1 Introduction

Acute respiratory distress syndrome (ARDS) is an acute clinical syndrome characterized by hypoxemic respiratory failure and bilateral pulmonary opacities resulting from diffuse inflammatory lung injury (Mane and Isaac, 2021). Despite lung-protective ventilation, prone positioning, and neuromuscular blockade, in-hospital mortality remains 30%–40% (Huang et al., 2020; Xie et al., 2025). According to the Berlin definition, ARDS is diagnosed when respiratory symptoms develop within 1 week of a known clinical insult or new or worsening respiratory symptoms, chest radiography or computed tomography shows bilateral opacities that are not fully explained by pleural effusions, lobar or lung collapse, or pulmonary nodules, and the respiratory failure is not fully explained by cardiac failure or fluid overload. When no clear risk factor for hydrostatic pulmonary edema is present, objective assessment, such as echocardiography, should be performed to exclude cardiogenic causes (Hanley et al., 2022). However, this clinical definition does not fully capture the substantial heterogeneity of ARDS in terms of etiology, underlying pathobiology, organ dysfunction, and treatment response (Matthay et al., 2020; Hendrickson et al., 2021; Al-Husinat et al., 2025).

ARDS may result from diverse direct pulmonary insults, such as pneumonia and aspiration, or indirect systemic insults, such as sepsis, trauma, and pancreatitis. These different initiating conditions can produce variable degrees of alveolar epithelial and endothelial injury, inflammation, coagulation disturbance, and impaired alveolar fluid clearance (Huang et al., 2024; Ziaka and Exadaktylos, 2025).

Accordingly, ARDS is increasingly regarded as a heterogeneous syndrome comprising biologically and clinically distinct subphenotypes rather than a single uniform disease entity (Lee et al., 2021; Wu et al., 2022). Among the proposed ARDS classifications, latent class analysis and related data-driven methods have consistently identified two major biological subphenotypes: a hyperinflammatory subphenotype, characterized by higher circulating inflammatory and endothelial-injury biomarkers, more severe organ dysfunction, and poorer outcomes; and a hypoinflammatory subphenotype, characterized by lower systemic inflammatory activity and generally better survival (He and Xu, 2021; Sinha et al., 2022). These subphenotypes may also differ in their responses to corticosteroids, neuromuscular blockade, positive end-expiratory pressure, and other interventions (Siuba et al., 2024).

Collectively, ARDS is a highly heterogeneous syndrome encompassing multiple subphenotypes with distinct biological, clinical, and therapeutic profiles. Integrating clinical data, biomarkers, omics, and machine learning offers opportunities for prognostic and predictive enrichment. However, translating these insights into routine practice requires further research, validation, and rapid diagnostic tools. This review systematically synthesizes current understanding of ARDS subphenotypes from systemic inflammatory biomarkers, prognostic implications, ventilatory management, and treatment, exploring the translational pathway toward precision medicine.

## 2 Pathobiological basis of ARDS heterogeneity and conceptual framework of subphenotypes

### 2.1 Alveolar–capillary barrier injury and mitochondrial dysfunction

Disruption of the alveolar–capillary barrier is central to ARDS pathobiology. This barrier comprises pulmonary microvascular endothelial cells, alveolar epithelial cells, and the extracellular matrix. Increased barrier permeability results in protein-rich pulmonary edema and impaired gas exchange. The relative contributions of epithelial injury, endothelial dysfunction, and local and systemic inflammation vary among patients and are influenced by direct pulmonary insults, such as pneumonia and aspiration, and indirect insults, such as sepsis and pancreatitis.

Molecular mediators discussed in relation to this injury include angiopoietin-2 (Ang-2), high-mobility group box 1 (HMGB1), and the receptor for advanced glycation end products (RAGE). Injury to alveolar epithelial type II cells (AECII) additionally compromises surfactant production and alveolar fluid clearance. Mitochondrial dysfunction provides another link between cellular injury and impaired barrier function, with AECII mitochondrial quality control discussed in relation to oxidative stress, inflammation, and fibrosis (Zeng and Yan, 2025). Mesenchymal stromal cell-derived extracellular vesicles have also been investigated for their ability to rescue mitochondrial dysfunction and improve barrier integrity in clinically relevant models of ARDS (Dutra Silva et al., 2021).

Mechanical ventilation may further aggravate epithelial and endothelial injury through ventilator-induced lung injury (VILI) (Meegan et al., 2024). Together, differences in barrier disruption, mitochondrial dysfunction, and inflammatory activity contribute to ARDS heterogeneity (Figure 1) and motivate investigation of strategies directed at barrier restoration and inflammatory regulation (Tie et al., 2025).

**FIGURE 1:**
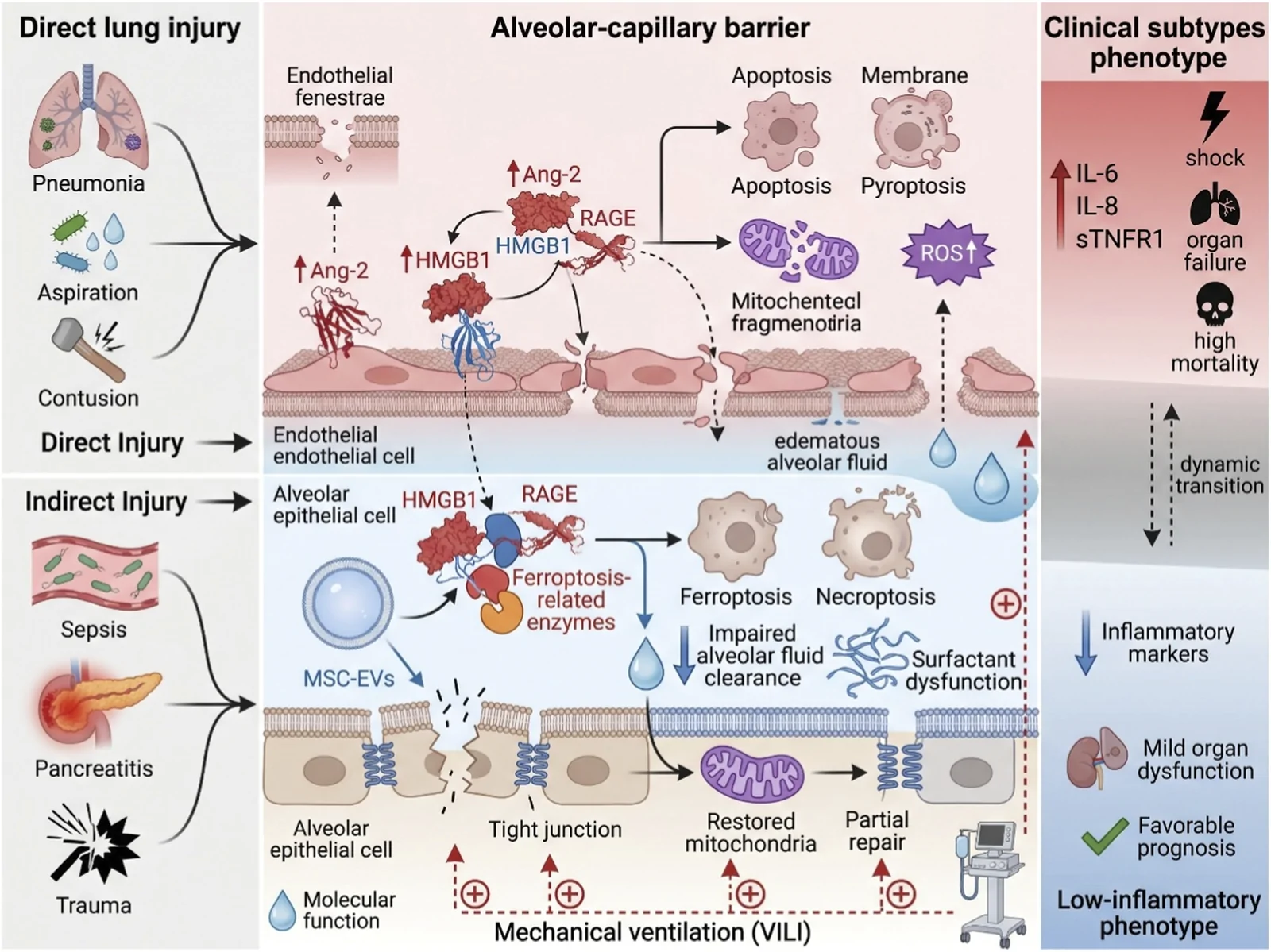
Alveolar-capillary barrier injury and inflammatory heterogeneity in ARDS. This schematic illustrates the disruption of the alveolar-capillary barrier following direct (e.g., pneumonia, aspiration) and indirect (e.g., sepsis) lung insults. Central to the figure are key molecular mediators, including Ang-2 and multiple HMGB family members, which highlight the quantitative and qualitative heterogeneity of damage-associated molecular patterns (DAMPs) driving endothelial and epithelial dysfunction. The resulting increased permeability, pulmonary edema, and impaired gas exchange are exacerbated by variable injury severity, differential activation of signaling pathways (e.g., RAGE, HMGB1, Ang-2), and mechanical ventilation-induced lung injury (VILI). This heterogeneity underlies clinical subphenotypes in ARDS and informs potential precision therapies targeting barrier restoration and inflammation.

### 2.2 Innate and adaptive immune dysregulation

Immune dysregulation in ARDS is heterogeneous, involving differences in cytokine release, immune-cell activation, and the balance of local and systemic inflammatory responses. Transcriptomic findings summarized in this review associate the hyperinflammatory subphenotype with innate immune activation, granulopoiesis, interferon signaling, T-cell activation, and integrated stress-response pathways. The hypoinflammatory subphenotype exhibits relatively greater expression of adaptive immune genes, particularly those related to T-cell function (Sinha et al., 2024; Sarma et al., 2025). These findings should not be interpreted as an exclusive separation of innate and adaptive immunity between the two subphenotypes.

Immune heterogeneity is also accompanied by metabolic differences. Plasma metabolic profiling links hyperinflammatory ARDS to dysregulated lipid metabolism and a glycolytic shift (Alipanah-Lechner et al., 2023). These observations support consideration of immune and metabolic processes together when exploring the biological basis of inflammatory subphenotypes.

Importantly, excessive cytokine release should not be assumed to occur uniformly across ARDS populations or biological compartments. Plasma-defined inflammatory subphenotypes do not consistently exhibit corresponding differences in alveolar inflammatory mediators, and BALF-derived classifications only partially overlap with plasma-based classifications (Heijnen et al., 2021; Sathe et al., 2023). Accordingly, discussion of a “cytokine storm” in ARDS should acknowledge heterogeneity in systemic and pulmonary inflammation rather than equating a plasma-defined hyperinflammatory state with uniformly increased alveolar inflammation.

### 2.3 Regulated cell death and candidate therapeutic targets

Regulated cell death is a further consideration in the pathobiology of ARDS. In addition to the involvement of ferroptosis noted in the original mechanistic overview, the literature cited in this review includes investigations of mesenchymal stem cell-derived extracellular vesicles targeting MAPK-mediated necroptosis to protect the alveolar epithelial barrier (Ruan et al., 2025), and a multifaceted nanomedicine strategy targeting PANoptosis and lung barrier injury (Cui et al., 2026). These studies identify cell-death regulation as a therapeutic research direction that complements approaches focused on circulating inflammatory biomarkers.

Ferroptosis has been investigated in several cellular compartments relevant to ARDS, including alveolar and bronchial epithelial cells, pulmonary endothelial cells, alveolar macrophages, and neutrophil-associated inflammatory networks. Its defining features include intracellular iron accumulation, phospholipid peroxidation, glutathione depletion, and dysfunction of the SLC7A11–GSH–GPX4 antioxidant system (Zheng et al., 2023). Other important regulatory mechanisms include ACSL4/ALOX15-dependent polyunsaturated fatty-acid oxidation, NCOA4-mediated ferritinophagy with FTH1 degradation, and activation of the NRF2/HO-1, STAT3, and IDO1 pathways (Chen Z. et al., 2024; Chen X. et al., 2024). In airway epithelial cells, ferroptosis is associated with mitochondrial injury, loss of E-cadherin, and impaired epithelial cohesion, whereas ferroptotic injury in pulmonary epithelial and endothelial cells can increase alveolar-capillary permeability and pulmonary edema (Xu et al., 2024). Ferroptotic damage may also release oxidized lipids and damage-associated molecular patterns, thereby amplifying neutrophil recruitment and inflammatory cytokine production (Chen Z. et al., 2024). In LPS-induced murine acute lung injury, ferrostatin-1 and liproxstatin-1 reduced lipid peroxidation, inflammation, pulmonary edema, and epithelial barrier disruption (Ma et al., 2023). Melatonin inhibited NCOA4-mediated ferritinophagy in alveolar macrophages and attenuated ferroptosis and lung injury in a septic ARDS mouse model. In influenza A virus-induced acute lung injury, ferrostatin-1 and the IDO1 inhibitor 1-methyl-tryptophan reduced epithelial ferroptosis and improved the lung injury phenotype. In another ARDS-specific study, reduced mast-cell-derived exosomal miR-744 promoted GPX4 loss and ACSL4/ALOX15 activation in airway epithelial cells, while miR-744 levels were also reduced in the plasma and exosomes of patients with ARDS (Zheng et al., 2025). Dipyridamole inhibited ferroptosis in epithelial and endothelial cells through the SOD1–CREB1–HMOX1 axis and showed protective effects in LPS- and cecal-ligation-and-puncture models, human airway organoids, and a small clinical proof-of-concept study (Fang et al., 2024). However, the clinical study included only five patients with moderate-to-severe ARDS and was not a randomized controlled trial, so the therapeutic efficacy of dipyridamole remains preliminary. Although ferroptosis-related genes have been associated with ARDS severity and neutrophil infiltration in bronchoalveolar lavage fluid, ferroptosis has not yet been validated as a biomarker-defined ARDS subphenotype or clearly linked to the established hyperinflammatory and hypoinflammatory phenotypes (Chen X. et al., 2024).

Pyroptosis in ARDS has been most consistently described in alveolar macrophages and recruited monocyte-derived macrophages, although pulmonary epithelial and endothelial cells may also undergo gasdermin-mediated inflammatory cell death (Wei et al., 2022). The canonical pathway involves TLR4–MyD88–NF-κB priming followed by activation of the NLRP3 inflammasome, ASC recruitment, and caspase-1 activation (Hu et al., 2024). The noncanonical pathway is initiated by intracellular LPS sensing through human caspase-4/5 or murine caspase-11 (Tao et al., 2026). Both pathways cleave gasdermin D, whose N-terminal fragment forms plasma-membrane pores and facilitates the release of mature IL-1β and IL-18 (Wu et al., 2023). Mitochondrial ROS, cytosolic mitochondrial DNA, and cGAS–STING–IRF3 signaling can provide upstream activation signals for macrophage pyroptosis (Li B. et al., 2025). The resulting cytokine release, DAMP liberation, and inflammatory-cell recruitment intensify epithelial injury, endothelial permeability, alveolar flooding, and disruption of the alveolar-capillary barrier. In LPS-induced ARDS models, 4-octyl itaconate suppressed mitochondrial ROS, cGAS–STING signaling, NLRP3 activation, GSDMD cleavage, and IL-1β/IL-18 release in alveolar macrophages. GC-1 inhibited ROS-mediated inflammasome assembly and pyroptosis through the Nrf2–p53–ASC pathway in LPS- or hydrochloric-acid-induced ALI models. Genetic deletion or siRNA-mediated inhibition of Bhlhe40 reduced both caspase-1-dependent and caspase-11-dependent GSDMD-mediated pyroptosis and alleviated LPS-induced lung injury. Dynasore also reduced macrophage pyroptosis by inhibiting the Drp1–ROS–NLRP3 axis *in vitro* and in LPS-induced murine ALI. Human evidence remains mainly associative: alveolar-macrophage transcriptomic datasets from patients with ARDS identified NLRP3, IL18, IL6, and GPX4 as genes associated with clinical outcome, while BALF caspase-4 levels in children with ARDS correlated with IL-1β, IL-6, and TNF-α (Liu et al., 2023). These patient studies support the clinical relevance of pyroptosis-related signaling but do not directly prove active pyroptotic cell death *in vivo*. No clinical trial has yet demonstrated that selective inhibition of pyroptosis improves ARDS outcomes, and pyroptosis has not been validated as a distinct ARDS subphenotype (Tao et al., 2026).

The relevance of these mechanisms to precision medicine requires a distinction between identifying a candidate molecular target and demonstrating benefit from a targeted intervention in a defined patient subgroup. As emphasized throughout this review, phenotype-guided therapeutic strategies require validated classification tools and prospective evaluation. Future studies integrating molecular measurements with clinical and alveolar data may help determine how cell-death-related mechanisms relate to established ARDS subphenotypes.

These pathobiological dimensions provide a framework for interpreting ARDS subphenotypes. Latent class analysis has identified inflammatory subphenotypes that are generalizable to observational ARDS cohorts (Sinha et al., 2022). Single-cell approaches provide complementary information on immune-cell heterogeneity and communication within the pulmonary compartment (Chen et al., 2025), while longitudinal multi-omics studies connect inflammatory phenotypes with molecular pathways associated with mortality (Alipanah-Lechner et al., 2026). Integrating these approaches may help relate biological heterogeneity to prognostic and therapeutic differences, but clinical implementation requires validated classification tools and prospective assessment of treatment response (Sinha et al., 2023a; Leonard and Sinha, 2024).

## 3 Hyperinflammatory and hypoinflammatory subphenotypes: the central biological framework

Among the available ARDS classification systems, hyperinflammatory and hypoinflammatory subphenotypes currently provide the most reproducible biological framework. This section therefore serves as the central part of the review and summarizes the principal circulating biomarkers, clinical characteristics, temporal behavior, prognostic implications, and treatment-response signals associated with these two subphenotypes.

### 3.1 Hyperinflammatory and hypoinflammatory subphenotypes

Latent class analysis (LCA) of plasma biomarkers (IL-6, IL-8, sTNFR1, Ang-2) dichotomizes ARDS into hyperinflammatory and hypoinflammatory subphenotypes. The hyperinflammatory subphenotype shows elevated biomarker levels, higher SOFA scores, more shock, and mortality >40%; the hypoinflammatory subphenotype has milder inflammation, less organ dysfunction, and better survival (Das et al., 2025; Neyton et al., 2024; R et al., 2025). These subphenotypes also occur in ventilated patients without ARDS, indicating broader relevance in critical illness (Drohan et al., 2021; Sinha et al., 2023b).

The immune and transcriptomic differences underlying these subphenotypes are discussed in Section 1.2 (Sinha et al., 2024; Sarma et al., 2025). Clinically, the hyperinflammatory subphenotype is associated with more severe organ dysfunction, with shock representing a prominent cause of death, whereas deaths in the hypoinflammatory subphenotype more often involve respiratory failure or comorbidities (Evrard et al., 2024).

Temporal analyses indicate that the hyperinflammatory phenotype is more prevalent early in critical illness but may decrease over time, with some patients transitioning to the hypoinflammatory phenotype—a shift associated with improved survival (Pensier et al., 2025; van Amstel et al., 2025). These subphenotypes have been validated across diverse populations, including pediatric ARDS cohorts (Yehya et al., 2024; Balcarcel et al., 2025). Notably, corresponding differences in bronchoalveolar lavage fluid (BALF) inflammatory mediators and lung microbiome composition are less pronounced, suggesting that systemic inflammation may not fully capture alveolar compartment biology (Heijnen et al., 2021; Sathe et al., 2023) (Table 1).

**TABLE 1:** Key differences between hyperinflammatory and hypoinflammatory ARDS subphenotypes.

Domain	Hyperinflammatory/Reactive subphenotype	Hypoinflammatory/Uninflamed subphenotype	Clinical implication	Ref.
Main identification approach	Latent class analysis or machine learning using plasma biomarkers and clinical variables	Same data-driven approaches, typically as comparator class with lower inflammatory activity	Reproducible and clinically relevant classification	(Matthay et al., 2020; Wu et al., 2022; Sinha et al., 2022; Hu et al., 2024; Tao et al., 2026; Wu et al., 2023; Li et al., 2025a; Liu et al., 2023; Goursaud et al., 2021)
Representative plasma biomarkers	Elevated IL-6, IL-8, sTNFR1, Ang-2, and other inflammatory/endothelial injury markers	Lower levels of inflammatory and endothelial injury biomarkers	Enables biological stratification and prognostic enrichment	(Sinha et al., 2022; Hu et al., 2024; Tao et al., 2026; Wu et al., 2023; Li et al., 2025a; Yusuff et al., 2022; Goursaud et al., 2021)
Clinical characteristics	Higher SOFA scores, more frequent shock, metabolic acidosis, greater extrapulmonary organ dysfunction	Less severe systemic inflammation, lower shock frequency, less organ dysfunction	Identifies patients with more severe systemic host response	(Sinha et al., 2022; Hu et al., 2024; Tao et al., 2026; Wu et al., 2023; Sinha et al., 2023a; Pensier et al., 2025)
Molecular and transcriptomic features	Upregulation of innate immunity, granulopoiesis, T-cell activation, interferon signaling, and integrated stress response pathways	Relatively greater expression of adaptive immune genes, particularly those related to T-cell function	Suggests distinct underlying immunopathological mechanisms	(Zeng and Yan, 2025; Tao et al., 2026; Chen et al., 2025; Alipanah-Lechner et al., 2026; Rafikov et al., 2025)
Prognosis	Higher mortality (often >40% in several cohorts); shock-related death more common	Better survival; deaths more often from respiratory failure or comorbidities	Provides prognostic enrichment for trials and bedside risk stratification	(Sinha et al., 2022; Hu et al., 2024; Tao et al., 2026; Wu et al., 2023; Sinha et al., 2023a; Pensier et al., 2025; van Amstel et al., 2025)
Temporal behavior	More prevalent early in critical illness; may persist or transition toward a hypoinflammatory state over time	May be present at baseline or emerge after transition from a hyperinflammatory state	Supports the need for dynamic reassessment rather than one-time classification	(Wu et al., 2023; Leonard and Sinha, 2024; Das et al., 2025)
Treatment response tendency	May benefit from corticosteroids, neuromuscular blockade, statins, or immunomodulatory strategies	Limited benefit or potential harm from indiscriminate anti-inflammatory/immunosuppressive therapies	Supports predictive enrichment and phenotype-guided therapy	(Siuba et al., 2024; Heijnen et al., 2021; Chen et al., 2025; Leonard and Sinha, 2024; Evrard et al., 2024; Goursaud et al., 2021; Mahida et al., 2022)
Major limitation	Plasma biomarkers primarily reflect systemic inflammation, not fully capturing alveolar compartment biology	Similarly may not reflect local pulmonary inflammation or microbiome-driven injury	Integration with BALF biomarkers, imaging, and omics needed	(Zhang et al., 2025; Dutra Silva et al., 2021; Drohan et al., 2021; Sinha et al., 2023b; Melamed et al., 2025; Nardelli et al., 2026)

### 3.2 Predictive enrichment and therapeutic implications of biomarker-based subphenotypes

The therapeutic relevance of inflammatory subphenotyping lies primarily in predictive enrichment. Retrospective analyses suggest that patients with hyperinflammatory features may have different responses to corticosteroids, neuromuscular blockade, statins, and other immunomodulatory strategies than patients with hypoinflammatory features (Siuba et al., 2024; Pensier et al., 2025). In contrast, indiscriminate anti-inflammatory or immunosuppressive treatment may provide limited benefit or potential harm in patients with a hypoinflammatory profile. These observations support the use of inflammatory subphenotypes as a framework for evaluating treatment heterogeneity. However, the available evidence does not establish a universal subtype-specific prescription rule, and prospective randomized trials are required before these classifications can be used to guide routine pharmacological treatment. Emerging therapies targeting alveolar barrier injury, mitochondrial dysfunction, ferroptosis, pyroptosis, necroptosis, and PANoptosis may complement inflammatory subphenotype-guided treatment. These interventions are biologically relevant to the inflammatory and tissue-injury processes observed in ARDS, but they have not yet been validated as treatments specifically directed at either the hyperinflammatory or hypoinflammatory subphenotype. Accordingly, they should be regarded as mechanism-based investigational strategies rather than established subtype-specific therapies.

The identification of hyperinflammatory and hypoinflammatory ARDS subphenotypes has profound implications for predictive enrichment and personalized therapy, as these subgroups exhibit differential responses to various treatments. Predictive enrichment refers to the ability of subphenotypes to forecast heterogeneous treatment effects, thereby guiding therapeutic decisions and optimizing clinical trial design. Retrospective analyses of randomized controlled trials (RCTs) suggest that hyperinflammatory patients may derive survival benefits from neuromuscular blockade, corticosteroids, and statins, whereas hypoinflammatory patients may experience no benefit or even harm (Pensier et al., 2025; Sinha et al., 2021).

The differential treatment responses are likely attributable to the underlying immunopathology. Hyperinflammatory patients exhibit excessive innate immune activation and endothelial injury, rendering them more amenable to immunomodulatory therapies that attenuate inflammation and vascular permeability. Conversely, hypoinflammatory patients, characterized by a more regulated immune response, may not benefit from such interventions and could be adversely affected by immunosuppression (Slim et al., 2024). This paradigm underscores the necessity of precise subphenotype identification to avoid indiscriminate treatment application that may exacerbate outcomes in certain patient subsets.

The temporal stability of these subphenotypes further informs therapeutic strategies. Studies employing Bayesian Markov models reveal that hyperinflammatory and hypoinflammatory phenotypes remain relatively stable over 28 days in ARDS patients, (Pensier et al., 2025). Importantly, corticosteroid therapy appears beneficial predominantly in patients who maintain a hyperinflammatory phenotype, with diminished or reversed effects in those transitioning to or remaining hypoinflammatory (Sinha et al., 2021). These findings advocate for dynamic monitoring of subphenotypes to tailor treatment duration and intensity.

To facilitate clinical translation, simplified bedside classification models using minimal biomarkers (angiopoietin-2, sTNFR1, procalcitonin, bicarbonate) achieve high accuracy and have been validated in independent cohorts, including pediatric populations (Balcarcel et al., 2025). Machine learning using electronic health record data further enhances feasibility by identifying subphenotypes without extensive biomarker panels. The integration of subphenotype classification into clinical trial design offers a pathway to precision medicine in ARDS. By enriching trials with patients most likely to respond to targeted therapies, such as hyperinflammatory individuals for immunomodulatory agents, statistical power is increased, and the likelihood of demonstrating treatment efficacy is enhanced. Moreover, the recognition of subphenotype-specific mortality attributable fractions informs sample size calculations and endpoint selection, addressing challenges in trial design posed by ARDS heterogeneity (Saha et al., 2023).

In summary, biomarker-based ARDS subphenotypes provide critical predictive enrichment that can guide therapeutic decisions and optimize clinical trial design. The development of simplified, validated classification tools is essential for bedside implementation, enabling precision medicine approaches that improve outcomes by aligning treatment strategies with underlying biological heterogeneity.

## 4 Subphenotypes based on pulmonary physiology and imaging

### 4.1 Respiratory mechanics and gas exchange-guided physiological subphenotypes

Machine learning integrating respiratory mechanics and gas exchange identifies distinct physiological subphenotypes. Key parameters—compliance, driving pressure, ventilation ratio, and mechanical power—serve as critical indicators of lung function. Unsupervised clustering reveals a “restrictive” subphenotype (low compliance, elevated driving pressure, impaired gas exchange) correlating with higher 28-day mortality and increased VILI risk.

In COVID-19 ARDS, upward trajectories in ventilation ratio or mechanical power predict higher mortality and fewer ventilator-free days, underscoring the importance of continuous monitoring over static assessment. Physiological subphenotypes also influence prone positioning responses; those with high pulmonary vascular permeability index and elevated ventilation ratio derive differential survival benefits from prone ventilation (Li L. et al., 2025).

Machine learning further refines subphenotype classification by integrating bedside ventilatory variables, accurately distinguishing “efficient” from “restrictive” subphenotypes. The efficient subphenotype associates with better gas exchange and lower mortality, highlighting the potential for real-time, data-driven stratification to personalize ventilation strategies (Meza-Fuentes et al., 2025).

In COVID-19 ARDS, meta-analyses show a normal distribution of compliance values without clear bimodal phenotypes, challenging discrete compliance-based classifications (Reddy et al., 2022). However, elevated dead space and V/Q mismatch due to microvascular thrombosis necessitate nuanced gas exchange assessment (Diehl et al., 2020). Driving pressure and mechanical power optimization improves outcomes, and reducing driving pressure to zero during ECMO may offer additional lung protection (Chiumello et al., 2025; Del Sorbo et al., 2020). Collectively, machine learning-facilitated physiological subphenotyping, continuous monitoring, and tailored ventilatory strategies provide a framework for precision medicine in ARDS.

### 4.2 Imaging morphological subphenotypes: focal and diffuse patterns

Chest computed tomography (CT) distinguishes focal from diffuse lung involvement in ARDS. The focal pattern shows gravity-dependent consolidation in posterior/basal regions, while the diffuse pattern exhibits homogeneous ground-glass opacities throughout both lungs, reflecting widespread alveolar involvement (Chotalia et al., 2021). These morphological distinctions have significant implications for respiratory mechanics and therapeutic responsiveness. Diffuse morphology demonstrates higher lung recruitability, with greater increases in ventilated lung tissue, compliance, and oxygenation following recruitment maneuvers or PEEP escalation (Wendel Garcia et al., 2021), whereas focal ARDS has lower recruitability due to consolidated, non-recruitable lung regions (Coppola et al., 2021).

The differential response to PEEP and prone positioning underscores their clinical relevance. Diffuse ARDS benefits from higher PEEP and prone positioning, which recruit collapsed alveoli and homogenize ventilation. In contrast, focal ARDS may experience increased lung stress and overdistension with high PEEP, and prone positioning yields less pronounced improvements (Coppola et al., 2021). These findings advocate for morphology-guided ventilatory strategies. Morphological subphenotypes also correlate with clinical etiologies: pneumonia often presents with focal consolidation, while sepsis-related ARDS typically shows diffuse involvement (Wendel Garcia et al., 2021). However, overlap with inflammatory biomarker-based subphenotypes is limited, indicating that imaging and biological markers capture distinct injury aspects.

Taken together, morphological subphenotyping into focal and diffuse patterns provides critical insights into ARDS heterogeneity. These subphenotypes differ in recruitability, respiratory mechanics, and responses to PEEP and prone positioning. When integrated with physiological and biological data, morphological classification enhances precision ARDS management (Table 2).

**TABLE 2:** ARDS subphenotypes based on respiratory physiology and imaging morphology.

Subphenotype	Main identifying features	Typical characteristics	Prognostic tendency	Implications for respiratory management	Ref.
Restrictive physiological subphenotype	Low respiratory system compliance, high driving pressure, increased mechanical power, elevated ventilation ratio, impaired gas exchange	Reduced lung distensibility, increased mechanical stress, greater VILI risk	Higher mortality, fewer ventilator-free days	Strict lung-protective ventilation; minimize driving pressure and mechanical power; close monitoring for deterioration	(Tie et al., 2025; Alipanah-Lechner et al., 2023; Sinha et al., 2021)
Efficient physiological subphenotype	Favorable respiratory mechanics and gas exchange profiles identified by machine learning using bedside ventilatory variables	Better gas exchange, lower mechanical burden, relatively preserved ventilatory efficiency	Generally better outcomes	Standard lung-protective ventilation appropriate; monitor for transition to higher-risk states	(Tie et al., 2025)
Dynamic high-risk physiological trajectory	Longitudinal increase in ventilation ratio, mechanical power, dead-space fraction, or worsening oxygenation	Progressive ventilatory inefficiency and V/Q mismatch	Increased mortality, prolonged mechanical ventilation	Repeated physiological assessment; timely adjustment of PEEP, tidal volume, prone positioning, or ECMO support	(Alipanah-Lechner et al., 2023; Yehya et al., 2024; Balcarcel et al., 2025; Slim et al., 2024)
Focal imaging subphenotype	CT: gravity-dependent, posterior, basal, lobar, or segmental consolidation	Lower recruitability; consolidated regions less responsive to recruitment maneuvers	Prognosis varies by etiology and severity	High PEEP may overdistend non-injured lung; recruitment strategies used cautiously	(Saha et al., 2023; Li et al., 2025b; Duan et al., 2026)
Diffuse imaging subphenotype	CT: homogeneous bilateral ground-glass opacities and diffuse consolidation	Higher recruitability, greater baseline elastance, widespread alveolar collapse	Often clinically severe but potentially more responsive to recruitment	May benefit from higher PEEP, recruitment maneuvers, and prone positioning if hemodynamically tolerated	(Saha et al., 2023; Li et al., 2025b; Duan et al., 2026)

## 5 Subphenotypes in specific clinical contexts and complications

### 5.1 Cardiovascular subphenotypes and right ventricular dysfunction

ARDS frequently coexists with circulatory failure. LCA of TTE and hemodynamic parameters identifies four cardiovascular subphenotypes: (1) normal ventricular function, (2) RV dilation with preserved systolic function, (3) RV dilation with systolic impairment, and (4) hyperdynamic left ventricular function. RV dilation with systolic impairment carries the highest 90-day mortality (78%), markedly exceeding conventional RV dysfunction definitions (Chotalia et al., 2023a). Similar findings in COVID-19 ARDS show mortality reaching 73% in the RV dilation with systolic impairment subgroup (Chotalia et al., 2022). RV systolic impairment correlates with hypoxemia and hypercapnia, while RV dilation associates with systemic organ dysfunction (Chotalia et al., 2021).

The pathophysiology involves increased RV afterload from pulmonary hypertension, hypoxic vasoconstriction, microthrombi, and elevated intrathoracic pressures, leading to septal shift and circulatory failure (Petit et al., 2021). Therapeutic implications include prone positioning, inhaled pulmonary vasodilators (e.g., nitric oxide), and ventilatory optimization to minimize driving pressures and hypercapnia (Fritzmann et al., 2025). Bedside echocardiography, including speckle-tracking strain imaging, enables early detection and monitoring of RV dysfunction (Temperikidis et al., 2022). In pediatric ARDS, RV dysfunction correlates with pulmonary hypertension and elevated brain-type natriuretic peptide (BNP) (Markiewitz et al., 2025). RV dysfunction can develop or worsen during VV ECMO, necessitating vigilant monitoring (Holton et al., 2024; Melamed et al., 2025). Emerging devices such as the ProtekDuo cannula offer percutaneous RV support (Nardelli et al., 2026).

### 5.2 ECMO-related subphenotypes and complication risks

Venovenous extracorporeal membrane oxygenation (VV ECMO) is an essential supportive therapy for severe ARDS refractory to conventional management, including COVID-19-related respiratory failure. Retrospective analyses have identified three ECMO-related subphenotypes based on pulmonary and systemic characteristics: “fibrotic,” “dry,” and “wet” (Chotalia et al., 2023b). The fibrotic subphenotype is characterized by higher propensity for lung fibrosis and is associated with a more protracted disease course and increased risk of adverse events. Notably, patients with the fibrotic subphenotype exhibit significantly elevated risk of intracranial hemorrhage (ICH), underscoring the need for vigilant anticoagulation management and neurological monitoring (Chotalia et al., 2023b). The increased bleeding risk may be multifactorial, involving ECMO-induced coagulopathy, endothelial dysfunction, and underlying fibrotic lung pathology.

Recognition of ECMO subphenotypes has important clinical implications. For the fibrotic phenotype, individualized anticoagulation protocols and frequent neurological assessments are required (Yusuff et al., 2022). Beyond bleeding risks, ECMO-related subphenotypes also differ in hemodynamic profiles and right ventricular function. RV dysfunction is common in ARDS patients on ECMO and associates with worse outcomes. Echocardiographic monitoring is essential to detect evolving RV dysfunction and guide therapy. In select cases, mechanical support devices such as the ProtekDuo cannula can provide targeted RV assistance (Nardelli et al., 2026).

ECMO-related subphenotypes may also influence ventilatory strategies. Ultraprotective ventilation with extracorporeal CO_2_ removal has been shown to improve RV function by reducing intrathoracic pressure and RV afterload (Goursaud et al., 2021). These subphenotypes are dynamic, requiring longitudinal assessment (Figure 2).

**FIGURE 2:**
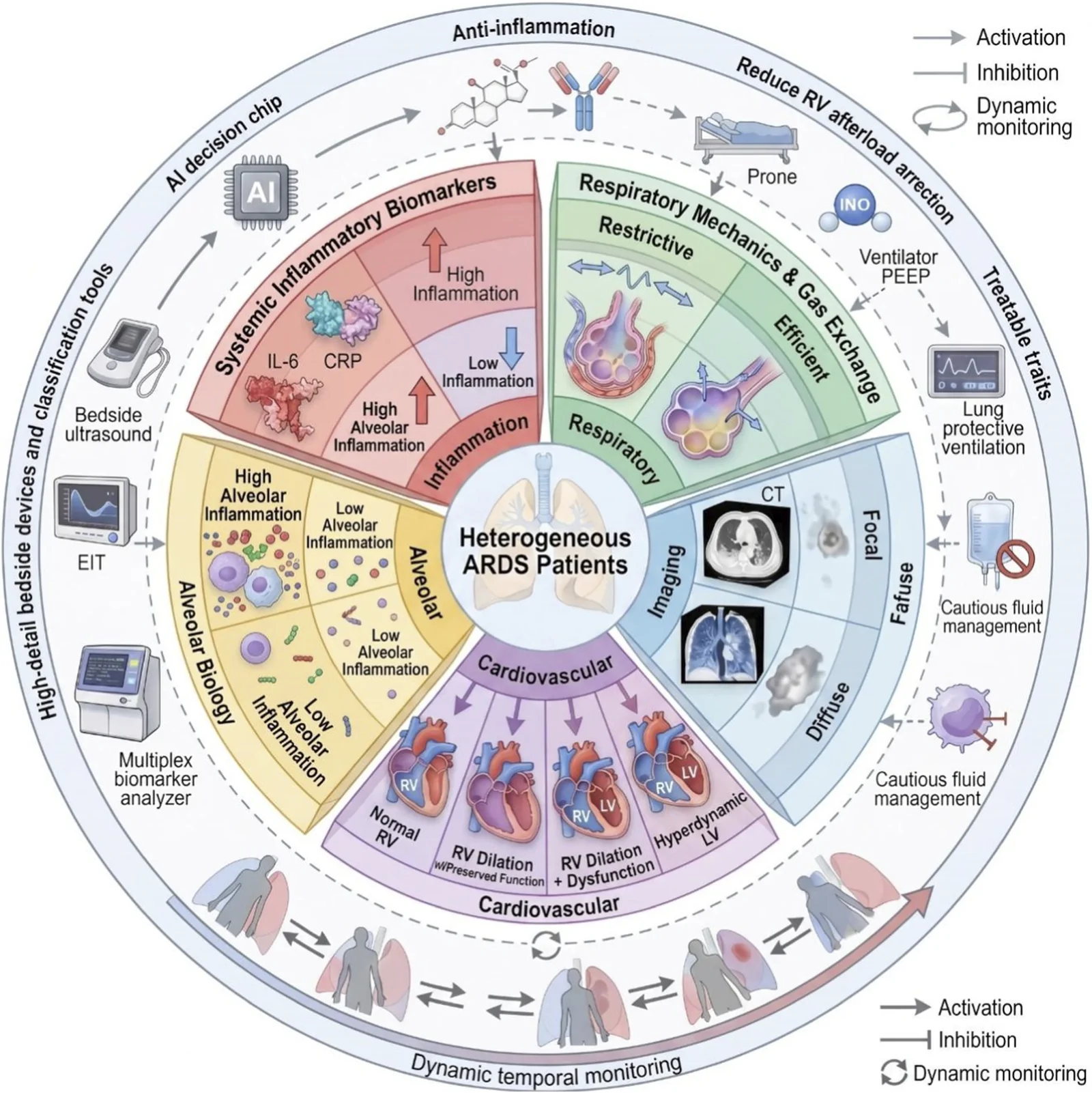
ARDS subphenotypes and phenotype-guided management. This schematic integrates inflammatory, physiological, imaging, and right ventricular (RV) subphenotypes of ARDS to guide precision therapy. The hyperinflammatory subphenotype (high IL-6/CRP, alveolar inflammation) may benefit from anti-inflammatory agents, whereas the hypoinflammatory subphenotype warrants cautious use. The restrictive physiological subphenotype (low compliance, high mechanical power) requires strict lung-protective ventilation, whereas the efficient subphenotype allows standard management. Diffuse imaging subphenotype (bilateral opacities) has high recruitability, favoring higher PEEP and prone positioning, while focal subphenotype (dependent consolidation) demands cautious PEEP. RV dilation with dysfunction necessitates fluid restriction and avoidance of high driving pressures. Bedside tools (EIT, lung ultrasound) and dynamic monitoring enable individualized, phenotype-guided treatment.

## 6 Disconnection between alveolar cavity biology and systemic inflammation

Alveolar biomarkers and lung microbiome data provide an important refinement of the systemic inflammatory framework. The incomplete concordance between plasma-defined subphenotypes and BALF profiles indicates that circulating biomarkers may not capture the full spectrum of local pulmonary inflammation. Consequently, some patients classified as hypoinflammatory on the basis of plasma markers may still have clinically relevant alveolar injury that requires pulmonary, rather than primarily systemic, treatment considerations.

### 6.1 Unique subphenotypes revealed by BALF biomarkers

Bronchoalveolar lavage fluid (BALF) biomarkers provide a more direct window into the pulmonary microenvironment than plasma markers, which predominantly reflect systemic inflammation. Studies analyzing BALF biomarkers such as IL-6, vWF, and SP-D have identified subphenotypes that only partially overlap with plasma-defined inflammatory subphenotypes. For example, latent class analysis of 14 BALF biomarkers revealed two distinct classes—one with elevated IL-6, vWF, soluble programmed cell death receptor-1(sPD-1), and neutrophil percentage, and another with lower levels—which correlated strongly with PaO_2_/FiO_2_ ratio and ventilatory parameters, unlike plasma subphenotypes that associated more with extrapulmonary dysfunction (Sathe et al., 2023).

Moreover, no consistent differences in alveolar inflammatory mediators exist between plasma-defined “hyperinflammatory” and “hypoinflammatory” subphenotypes; a study measuring multiple cytokines in mini-BAL fluid found no significant differences between “uninflamed” and “reactive” plasma subphenotypes (Heijnen et al., 2021). This decoupling suggests that therapies targeting systemic inflammation may not adequately address alveolar injury. Elevated CD14^+^/CD81^+^ extracellular vesicles in BALF associate with sepsis-related ARDS severity and mortality, highlighting their potential as biomarkers of local immune dysregulation (Mahida et al., 2022).

### 6.2 Role of the lung microbiome in subphenotypes

The lung microbiome further influences local immune responses and alveolar injury. Alterations—including increased bacterial burden, reduced diversity, and gut-associated bacterial translocation—exacerbate lung inflammation. However, plasma-defined “hyperinflammatory” subphenotypes do not consistently exhibit distinctive lung microbiome signatures; a study found no significant differences in microbiome features between “uninflamed” and “reactive” plasma subphenotypes. The lack of concordance between plasma subphenotypes and lung microbiome profiles highlights a limitation of current classification systems that rely predominantly on systemic markers. Since the lung microbiome directly interacts with alveolar immune cells, its dysbiosis likely drives localized inflammatory cascades not fully mirrored in peripheral blood. Integrating lung microbiome data with alveolar biomarker profiles could enhance subphenotype resolution. For example, increased bacterial load and reduced microbial diversity associate with heightened inflammation and worse outcomes, and gut-associated bacterial translocation may further exacerbate local injury (Duan et al., 2026).

Refining ARDS subphenotypes by incorporating BALF and lung microbiome data has significant therapeutic implications. Current trials targeting systemic inflammation may fail in patients whose primary pathology is driven by alveolar inflammation or microbial dysbiosis. Tailoring interventions to lung-specific biology—such as personalized ventilatory strategies guided by BALF profiling or antibiotics/probiotics aimed at restoring microbial homeostasis—could improve efficacy. In summary, the disconnect between systemic inflammation and alveolar biology underscores the need to integrate alveolar compartment and microbiome data into ARDS phenotyping frameworks. Such integration will enhance understanding of heterogeneity and facilitate development of precision therapies targeting lung-specific pathobiological processes.

## 7 Challenges and pathways for translating subphenotypes into precision medicine

### 7.1 Development and validation of bedside operable classification tools

The translation of complex research-level subphenotype models into clinically practical, bedside-applicable tools remains one of the most formidable challenges in ARDS management. Ideal classification tools must be rapid, cost-effective, and straightforward enough to be implemented at the bedside without requiring extensive resources or time (Maurer et al., 2021). This necessity arises from the clinical imperative to stratify patients promptly to guide precision therapies tailored to distinct ARDS subphenotypes. Current research efforts have focused on developing parsimonious models that rely on a minimal set of clinical variables and biomarkers, thereby enhancing feasibility and scalability in diverse clinical settings. For example, a recent study introduced the “HAIS score,” which utilizes only two biomarkers—interleukin-8 (IL-8) and soluble ST2 (sST2)—to identify a high-inflammation subphenotype among ARDS patients receiving nasal high-flow oxygen therapy (Zalucky et al., 2024). However, the clinical utility of such models hinges on rigorous validation in independent, multicenter prospective cohorts to ensure reproducibility, accuracy, and stability over time and across populations. Validation studies must also assess the concordance of these simplified tools with established subphenotype classifications and their predictive value for treatment response (Sinha et al., 2023a). The integration of artificial intelligence (AI) and machine learning (ML) methodologies offers promising avenues for developing interpretable, nonlinear risk calculators that can be embedded into user-friendly interfaces such as smartphone applications, facilitating dynamic bedside decision-making (Leonard and Sinha, 2024).

### 7.2 Prospective clinical trial design and therapeutic target matching

A pivotal future direction in ARDS research is the design and execution of prospective randomized controlled trials (RCTs) that test therapeutic strategies targeted to specific subphenotypes identified by validated classification tools. This approach necessitates stratifying patients at enrollment based on their subphenotype, followed by random allocation to either targeted interventions or standard care controls. Such stratification ensures that treatment effects are evaluated within biologically and clinically homogeneous groups, thereby enhancing the likelihood of detecting meaningful benefits that might be obscured in heterogeneous populations (Nazarian et al., 2026). Potential “treatable traits” in ARDS include immunomodulatory therapies directed at the high-inflammation subphenotype, such as cytokine antagonists targeting IL-6 or IL-1 pathways. Candidate interventions directed at cellular injury or regulated cell death should likewise be evaluated in relation to the underlying biological context, rather than assumed to benefit all patients within a broadly defined inflammatory subphenotype. Additionally, personalized mechanical ventilation strategies tailored to physiological subphenotypes—such as individualized PEEP settings guided by lung morphology—represent another critical therapeutic avenue. For instance, morphology-guided ventilation differentiates between focal and nonfocal ARDS patterns, which respond differently to recruitment maneuvers and PEEP titration. Furthermore, anticoagulation strategies adjusted according to coagulation dysfunction subphenotypes may optimize outcomes by addressing the prothrombotic milieu characteristic of certain ARDS subsets. Trial designs must account for the dynamic nature of ARDS subphenotypes, recognizing that patients may transition between phenotypes during the disease course. Advanced monitoring techniques, including echocardiography and esophageal manometry, can facilitate real-time phenotyping and guide individualized therapy adjustments. The integration of these dynamic, phenotype-stratified approaches into RCTs represents a paradigm shift toward precision medicine in ARDS (Chalkias et al., 2025) (Figure 3).

**FIGURE 3:**
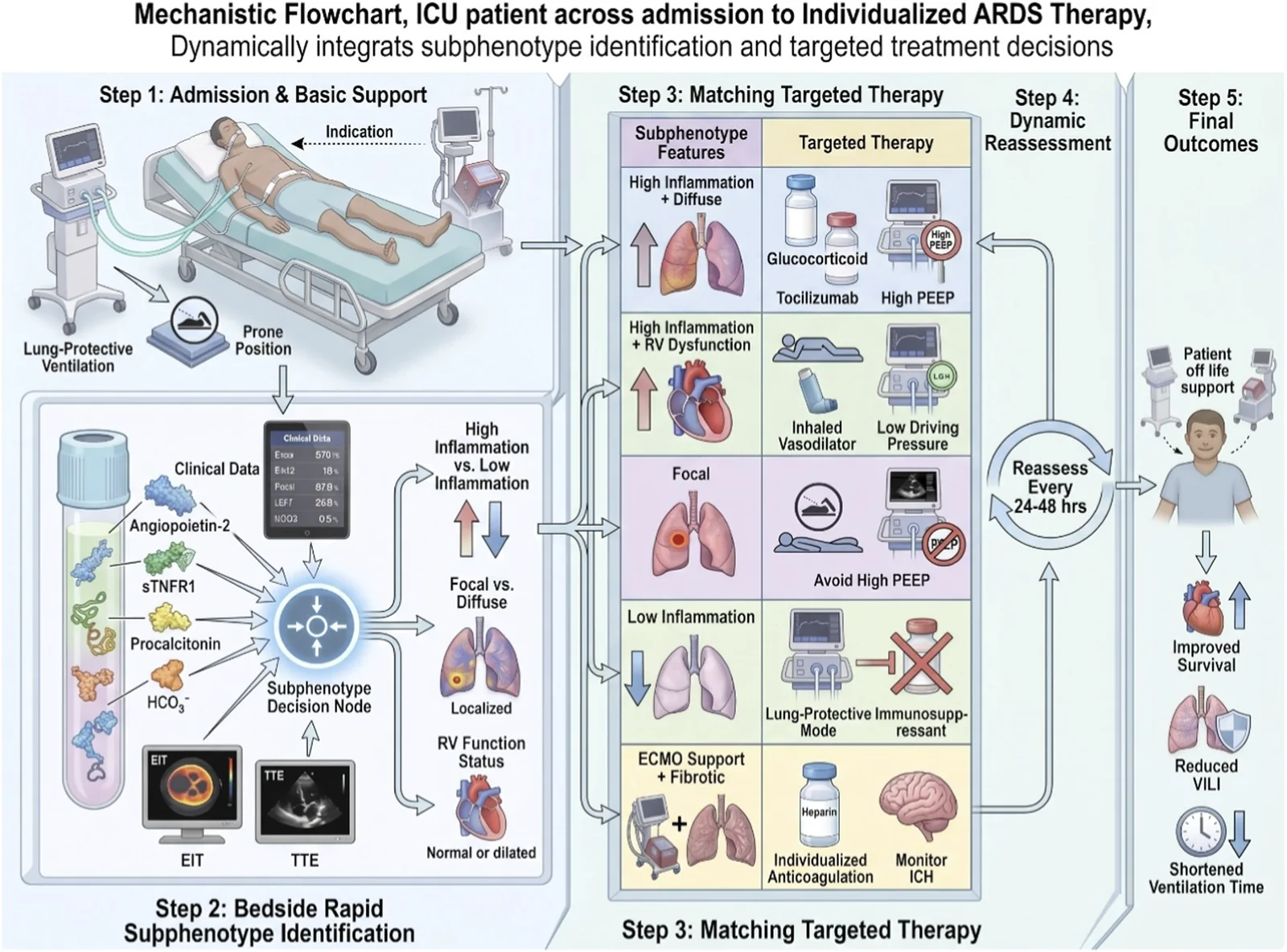
A dynamic algorithm for translating ARDS heterogeneity into precision medicine.

This flowchart illustrates a stepwise, bedside framework that integrates subphenotype identification with targeted therapy in ARDS. Following admission and basic supportive measures (lung-protective ventilation, prone positioning), clinical and biomarker data (Ang-2, sTNFR1, PCT, HCO_3_^−^) combined with bedside tools (EIT, TTE) inform rapid subphenotype classification. Patients are stratified by inflammatory status (high vs. low), morphological pattern (focal vs. diffuse), and right ventricular (RV) function (normal vs. dilated/dysfunctional). High-inflammation/diffuse phenotypes may benefit from glucocorticoids, tocilizumab, or high PEEP, whereas focal phenotypes require low driving pressure and avoidance of high PEEP. RV dysfunction prompts inhaled vasodilators, fluid restriction, and reduced driving pressure. ECMO, anticoagulation, and immunosuppressants are reserved for refractory or fibrotic cases. Dynamic reassessment every 24–48 h guides ongoing adjustments, aiming to shorten ventilation time, reduce VILI, and improve survival.

## 8 Future directions and cross-disease spectrum expansion

### 8.1 Multimodal data integration and advanced applications of artificial intelligence

The future of ARDS subphenotyping lies in integrating diverse data modalities—clinical parameters, multi-omics biomarkers (genomics, transcriptomics, proteomics, metabolomics), continuous physiological monitoring, and high-dimensional imaging. This multimodal fusion is essential to capture ARDS heterogeneity that single-modality analyses cannot fully elucidate. Recent studies demonstrate the feasibility of multimodal predictive models combining clinical data with advanced imaging and deep learning. For instance, integrating radiomics from three-dimensional CT with clinical variables and convolutional neural networks has enabled early ARDS prediction in acute pancreatitis patients, outperforming traditional scoring systems (Zhou et al., 2026). Prospective cohorts like the BIOWARE study are collecting longitudinal clinical, ventilator waveform, imaging (CT, EIT, lung ultrasound), and biospecimen data to identify biologically grounded ARDS endotypes through multimodal phenotyping (Tan et al., 2026).

Artificial intelligence, particularly deep learning, plays a pivotal role in processing these high-dimensional datasets. AI models uncover intricate, nonlinear relationships and latent patterns, facilitating discovery of more refined and clinically relevant subphenotypes. Multimodal AI frameworks integrating clinical, metabolomic, and biomarker data have demonstrated superior predictive accuracy for ARDS mortality, revealing metabolic pathways (e.g., tryptophan-kynurenine, NAD + metabolism) that underpin disease severity (Rafikov et al., 2025). AI-driven multimodal imaging analysis combining CT and EIT data has enhanced ARDS severity stratification and provided insights into ventilation/perfusion mismatches critical for personalized ventilatory management (Jiang et al., 2025).

The ultimate goal is constructing dynamic, digital phenotypes that characterize ARDS heterogeneity and predict individual treatment responses in real time, enabling precision medicine tailored to a patient’s evolving physiological and molecular profile. aAdvances in AI methodologies—graph neural networks, causal inference, federated learning, and large language models—promise to further enhance multimodal integration, interpretability, and clinical applicability (Li S. et al., 2025). However, challenges remain in data standardization, model generalizability, and ethical considerations, necessitating collaborative efforts to develop robust, transparent, and clinically validated AI tools.

### 8.2 Universality of ARDS subphenotype concepts in critical care

Emerging evidence suggests that biologically defined subphenotypes, particularly hyperinflammatory and hypoinflammatory profiles, are not exclusive to ARDS but prevalent across a broad spectrum of critical illnesses. These subphenotypes have been identified in patients with acute respiratory failure from diverse etiologies and even in critically ill patients without primary respiratory pathology, suggesting they represent fundamental host response patterns to severe systemic stress rather than disease-specific phenomena (Muñoz et al., 2026). Integrative multi-omics analyses reveal that hyperinflammatory ARDS phenotypes exhibit enhanced innate immune activation, metabolic reprogramming, and mitochondrial dysfunction—features also observed in sepsis and other critical conditions (Alipanah-Lechner et al., 2026).

Recognizing this cross-diagnostic applicability opens new avenues for precision medicine in critical care, shifting from syndrome-based classifications to biology-driven stratification. Treatments modulating inflammation or metabolic pathways could be selectively applied to patients exhibiting the hyperinflammatory phenotype, regardless of underlying critical illness (Deng et al., 2025). Future research must focus on delineating the distribution, temporal stability, and therapeutic implications of these subphenotypes across heterogeneous critical care cohorts. Large-scale, multicenter studies integrating multimodal data are essential to validate subphenotype classifications and assess their predictive value for treatment response. Adaptive clinical trial designs incorporating subphenotype stratification could accelerate evaluation of targeted therapies, fostering a shift toward “treatable traits” in critical care medicine (Muñoz et al., 2026).

## 9 Conclusion

ARDS remains a formidable challenge in critical care due to its marked biological and clinical heterogeneity, which has long hindered the development of universally effective therapies. The emergence of subphenotyping, particularly the reproducible distinction between hyperinflammatory and hypoinflammatory profiles based on biomarkers, physiology, and imaging, has provided a transformative framework for prognostic and predictive enrichment. Retrospective evidence demonstrates that these subphenotypes exhibit differential responses to interventions such as corticosteroids, prone positioning, and PEEP titration, underscoring the potential for phenotype-guided precision therapy. However, the translation of these insights into routine bedside practice requires the development of rapid, validated, and operationally simple classification tools, alongside prospective randomized controlled trials to confirm treatment efficacy within defined subgroups.

Beyond ARDS, the recognition that inflammatory subphenotypes are prevalent across diverse critical illnesses points to a common host response framework that transcends traditional diagnostic boundaries. The dynamic nature of subphenotypes, coupled with the often incomplete concordance between systemic inflammation and local alveolar biology, further emphasizes the need for multimodal data integration, longitudinal reassessment, and advanced analytical approaches such as artificial intelligence. Achieving precision medicine in ARDS will demand multidisciplinary collaboration, rigorous validation, and a sustained commitment to translating biological heterogeneity into actionable clinical strategies, ultimately improving outcomes for patients with ARDS and other critical conditions.
